# Match running performance upon return to play in professional male LaLiga football players following anterior cruciate ligament rupture

**DOI:** 10.5114/biolsport.2026.160857

**Published:** 2026-04-13

**Authors:** Manuel Manchón-Davó, Aarón Miralles-Iborra, Juan Del Coso, Francisco J Vera-Garcia, Heidy Rondón-Espinosa, Casto Juan-Recio, Joaquín González-Rodenas, Roberto López Del Campo, Ricardo Resta, Víctor Moreno-Pérez

**Affiliations:** 1 Sports Research Centre (Department of Sport Sciences), Miguel Hernandez University of Elche, Alicante, Spain; 2 Sport Sciences Research Centre, Rey Juan Carlos University, Fuenlabrada, Spain; 3 Institute for Health and Biomedical Research (ISABIAL Foundation), Miguel Hernández University of Elche, Alicante, Spain; 4 Real Madrid Graduate School, Universidad Europea de Madrid, 18071 Madrid, Spain; 5 Department of Competitions, La Liga, 28043 Madrid, Spain; 6 Translational Research Centre of Physiotherapy, Department of Pathology and Surgery, Faculty of Medicine, Miguel Hernandez University, Alicante, Spain

**Keywords:** Knee injury, Soccer, Elite athlete, Return to competition, Career length

## Abstract

This study aimed to investigate the impact of an anterior cruciate ligament rupture on the level of competition and match running performance over the following three seasons after return-to-play in professional male football players. Fifty-one football players from LaLiga who sustained a complete anterior cruciate ligament rupture were retrospectively followed over the three seasons after their return-to-play. Their level of competition and match running performance metrics were obtained via Mediacoach^®^ and subsequently compared across different time points: (1) PRE (season before injury); (2) INJ (season of the anterior cruciate ligament rupture); and (3) POST1, (4) POST2, and (5) POST3 (first, second, and third seasons after returning-to-play). Outcomes were analysed overall, by field position and by age group (≤ 25 and > 25 years). By the POST3 season following the anterior cruciate ligament injury, 35 of the 51 players (68.5%) were still competing in one of the top five UEFA leagues (34 in LaLiga and 1 in another top-five league), 11 (21.6%) were playing in lower-tier leagues, and 5 (9.8%) had retired. Maximum running speed decreased at POST1 and POST2 (p < 0.050) compared with PRE. Only players > 25 years experienced a significant decrease in their maximum running speed during POST2 and POST3 (p < 0.050) in comparison with PRE-values. A complete anterior cruciate ligament rupture in football from LaLiga players led to a decline in the level of competition and reductions in their maximum running speed up to three seasons after return-to-play. Players > 25 years were more vulnerable to sustain performance losses.

## INTRODUCTION

Anterior cruciate ligament (ACL) rupture occurs at an incidence of 0.0016 injuries per 1000 hours of play in football (soccer), corresponding to approximately 11 cases per season in Spain’s top professional league (*LaLiga* [1]). ACL rupture rates in football have not decreased in recent years [2] despite efforts by professional teams to reduce the incidence of this injury and the recent implementation of comprehensive prevention strategies targeting biomechanical risk factors through optimization of movement patterns across the kinetic chain [3]. ACL rupture is considered the most severe injury, and it is the injury with the highest burden among professional football players [4, 5] and it is associated with early-onset knee osteoarthritis [6] and a greater chance of suffering a reinjury [4, 7].

Generally, it is well known that injuries involve a period of inactivity that leads to physical detraining [8, 9]. In this sense, previous studies have found that detraining in football players leads to a reduction in the maximal aerobic speed [10], an impairment of the maximal oxygen consumption [11], a blood haemoconcentration process [10], increases in the percentage of fat-mass [10, 12] and a decrement in lean-mass or fat-free mass [12]. According to the literature [8], the extent of detraining will depend on the magnitude and duration of inactivity. Specifically, the prolonged recovery following an ACL rupture, averaging 218 to 248 days before returning to play, represents a significant detraining period [1, 13, 14]. This produces a high burden for football teams, which has negative economic implications, also affecting the team and player performance during competition.

In this regard, previous studies have investigated players’ performance upon return to play (RTP) following an ACL reconstruction in football players [7]. Interestingly, although previous studies in professional male football players reported very high rates of RTP at the preinjury competitive level one year after an ACL reconstruction [4, 15–17], only 50–65% of players were still competing at that level 3–4 seasons post-surgery [4, 14]. Additionally, evidence suggests that age and the playing position on the field may influence the degree of performance decline upon RTP following an ACL rupture [7, 14, 16, 18]. For instance, previous studies [7, 14] reported that players > 30 years presented higher rates of career termination or transfer to lower-level leagues after their injury. In this regard, Mazza et al. [14] reported, in a sample of 183 football players, that those younger than 25 years showed a significant reduction in minutes played per season only during the first postoperative season, whereas this decline persisted for up to three seasons after return to play in players older than 25 years. Similarly, Balendra et al. [18] in a cohort of 215 football players that those younger than 25 years had a higher rate of return to play in professional football compared with older players. In contrast, findings on the impact of playing position remain inconsistent, with some studies reporting positional differences while other studies do not report such differences [14, 19]. For example, Mazza et al. [14] reported no significant differences in postoperative playing time among positions, while Forsythe et al. [19] suggested that attackers may require longer to fully recover their pre-injury performance levels, including minutes, matches, goals, and assists. Collectively, these findings indicate that long-term recovery and performance after ACL reconstruction are influenced by player-specific factors such as age and position; however, the extent of these effects on players’ careers in elite-level competitions, such as *LaLiga*, remains unclear and warrants further investigation.

Beyond the effect on players’ career trajectories, previous studies [14, 16, 17, 19, 20] and a recent systematic review [21] have also examined the impact of an ACL rupture on on-field performance metrics upon RTP [14, 16, 17, 19, 20]. Several of these studies observed a significant reduction in the mean number of goals scored, minutes played, and matches participated in per season during the two to five seasons following RTP [14, 16, 17, 19, 21]. This may indicate that, although players are able to return to professional football, their participation and role within the team are often diminished in the seasons following RTP. Despite the growing body of research focused on performance outcomes such as scored goals, minutes played and matches participated in [21] the impact of an ACL rupture on match running performance parameters (e.g., total distance covered, sprint distance covered, number of sprints, and maximum speed) in the seasons following RTP remains poorly investigated, particularly in the long term. It is well established that match running performance is a crucial component of football because it directly affects a player’s ability to perform, contributes to overall team success, and may even influence market value. Additionally, recent studies have emphasized the importance of individualized rehabilitation strategies and on-field monitoring technologies, such as GPS tracking, suggesting that GPS-monitored on-field rehabilitation programs may produce a high chance of returning to competition post-ACL reconstruction [22, 23]. However, these investigations focus primarily on the rehabilitation process and short-term return to play, leaving a gap in the literature regarding the long-term evolution of match running performance once players have reintegrated into elite competition.

Understanding the impact of an ACL injury on match running performance parameters upon RTP, and in the subsequent seasons, is crucial to ensure adequate physical fitness, optimize performance, and reduce the risk of reinjury [23]. To the best of our knowledge, this is the first study to longitudinally assess the effects of an ACL rupture on both the level of competition and detailed match running performance metrics over three consecutive seasons after RTP in professional male players from *LaLiga*. A secondary aim was to explore its impact according to field positions and players’ age. Based on previous studies reporting reductions in playing minutes and the number of matches upon RTP after an ACL injury [14, 16, 17, 19], we hypothesised that several match-running performance metrics would decline following their RTP, particularly in older players and forwards, but would gradually improve over subsequent seasons.

## MATERIALS AND METHODS

### Sample size calculation

An *a priori* sample size estimation was conducted using G*Power. Because G*Power does not provide a direct non-parametric option, the sample size was calculated based on the methodological parametric equivalent of the Friedman test, namely a repeated-measures ANOVA (within-subject factors). Assuming a medium effect size (f = 0.25) of ACL injury on maximal running speed across the seasons after RTP, an alpha level of 0.05, a statistical power of 0.80, five repeated measures, a correlation of 0.6 among repeated measures and a non-sphericity correction of 1, the required sample size was 18 players. In order to maximize the statistical power of the analysis, we included all the players who met the inclusion criteria.

### Participants

A total of 51 male football players with a primary ACL rupture were included and retrospectively followed. All the football players competed in *LaLiga* and had sustained an ACL rupture between the 2011–2012 and the 2020–2021 seasons. Inclusion criteria were as follows: (a) age over 18 years; (b) belonging to the club’s first team; (c) ACL injury sustained during football-related activities (training or match play); and (d) availability of data on playing status for the season before the injury, the season in which the injury occurred and up to three seasons after RTP, regardless of whether the player remained in *LaLiga*, was transferred to another league or retired. The total sample was analysed to determine career trajectory and competitive level up to three seasons after RTP. However, match running performance was assessed only in a subset of 34 players, as these were the ones who were still playing in *LaLiga* three years after RTP, and Mediacoach^®^ data were only available for this group. Complete match running metrics for the season before the injury, the season of the injury, and up to three seasons after RTP was an additional inclusion criterion required for this subset. Goalkeepers were excluded from the performance analysis as their positional role entails markedly different physiological and match-running demands compared with outfield players [24–26]. All data was anonymised according to the Declaration of Helsinki to ensure player and team confidentiality. This study uses only publicly available data and, therefore, does not require informed consent from the players. The Institutional Review Board of the University approved this study (code: DPC.VMP.240213). Furthermore, *LaLiga* authorised the utilisation of the data about match running performance metrics for the present study.

### Injury and RTP definitions

According to a previous study, any ACL rupture included in the data analysis was considered as *“a first-time total rupture of the ligament occurring either isolated or associated with other concomitant”* [27]. In the present study, all included cases met this definition, that is, all were first-time ACL ruptures, surgically confirmed, and included regardless of the presence of additional associated injuries such as meniscal or collateral ligament damage. Furthermore, all the injuries were analysed irrespective of the mechanism (e.g., direct contact, non-contact or indirect contact ACL injuries). The term RTP was defined as *“having played at least one competitive game (irrespective of the minutes on the field) after the ACL reconstruction”* [14].

### Players’ data and career follow-up

Player and ACL rupture–related data were collected, including: (a) height; (b) body mass; (c) body mass index (BMI); (d) injured leg; (e) whether the injury occurred in the dominant leg or not; (f) playing position (defender, midfielder, or forward); (g) age at the moment of the injury (≤ 25 or > 25 years) based on Mazza et al. [14]; (h) injury context (friendly match, competitive match, or training); (i) time to RTP (as the number of days between the date of the ACL rupture and the date the player participated in their first official match following surgical treatment); and (k) total minutes played per season [14].

For the classification of competition level at RTP, players were grouped as follows: (a) “Top 5 UEFA leagues” if the player was registered in the first team of a club competing in one of the top 5 UEFA leagues (*LaLiga* [Spain], Serie A [Italy], Bundesliga [Germany], Premier League [England] and Ligue 1 [France]); (b) “Lower-level league” if the player was transferred to any other club outside the Top 5 UEFA leagues [14]; and (c) “Retired” if the player ceased professional competition for any reason (no official appearances in professional leagues). Each player was categorised according to this classification based on the United European Football Association (UEFA) country rankings for every season up to three seasons after RTP [14]. Transfers and season-by-season registration were verified from official league registrations, official club rosters and press releases, public databases (e.g. Transfermarkt.es) and, when available, direct information from the teams’ staff. The data was corroborated by at least 2 independent sources of information. Match running performance data were provided by *LaLiga* through the Mediacoach^®^ system. All these data were obtained for the whole sample of 51 players and categorised as one season before the injury (hereinafter referred to as PRE), the season of the injury (hereinafter referred to as INJ) and up to three seasons after players’ RTP (hereinafter referred to as POST1, POST2 and POST3, respectively). All these parameters were collected and manually cross-referenced independently by two authors (M.M.D. and V.M.P.).

### Match running performance metrics

The following match running performance metrics were obtained by the multicamera Mediacoach^®^ system based on the OPTA^®^ (Spain) track analysis tool: (a) total minutes played or match exposure (min); (b) total distance covered per minute (m/min); (c) distance covered at sprinting (sprints were considered those displacements exceeding 24 km/h, according to standard threshold used by Mediacoach^®^) per minute (m/min); (d) number of sprints per minute (n/min); and (e) maximum speed of the season (km/h). Mediacoach^®^ is used by Spanish football teams to evaluate running performance indicators in competition [28] and has recently demonstrated very good agreement against GPS data for total distance, distance at > 24 km/h, number of sprints above 24 km/h, and maximum speed [29, 30]. In addition, the validity of the injury-related data retrieved from Transfermarkt.es has been analysed, reporting values corresponding to a Cohen’s kappa of 0.82 for the cross-validation, and the reliability measurement of injury type/localisation was 89% [31]. All the match running performance metrics were recorded for the subset of 34 players who remained in *LaLiga* for up to three seasons after RTP. Metrics were collected for the same seasons used in the career follow-up (PRE, INJ, POST1, POST2, and POST3) for each player in this subset. All the parameters were independently collected and manually cross-verified by two authors (M.M.D. and V.M.P.).

### Statistical analysis

Statistical analysis was conducted using custom-made scripts in Python 3.7, employing open-source libraries: NumPy v1.12.1. [32], SciPy.stats v1.13.1 [33] and Pingouin v0.5.5 [34]. Continuous variables were reported as mean ± standard deviation [SD] or median ± interquartile range (IQR), while categorical variables were reported as absolute numbers and percentages of the total sample. Demographic values were provided for all the ACL injuries (n = 51). Furthermore, the level of competition of 51 injured players was shown overall and stratified by age (≤ 25 and > 25 years), and playing field position (defenders, midfielders, and forwards) [14]. The χ^2^ test was used to compare competition level (top 5 UEFA elite leagues, lower league or retired) across the INJ, POST1, POST2 and POST3 seasons. In addition, the level of performance of the 34 injured players that continued in *LaLiga* during POST1, POST2 and POST3 was provided overall, grouped by age (≤ 25 and > 25 years) and grouped per field position (defenders, midfielders, and forwards) [14]. As the assumptions of normality and sphericity were not met, the Friedman test, a non-parametric alternative to repeated-measures ANOVA, was used to compare running performance parameters across all seasons (PRE, INJ, POST1, POST2 and POST3). Pairwise post-hoc comparisons using Wilcoxon signed-rank tests were conducted when the Friedman test indicated significant differences. p-values were adjusted for multiple testing using the Bonferroni correction. Confidence intervals (CI) for pairwise comparisons were calculated at the 95% level using a non-parametric bootstrap resampling procedure with 1000 iterations, defined by the 2.5^th^ and 97.5^th^ percentiles of the resampled distribution. All the variables included in the analyses were complete, with no missing values requiring imputation or exclusion. A statistical significance threshold of p < 0.050 was applied a priori to all statistical tests.

## RESULTS

Overall, 51 primary ACL ruptures were included, with players presenting a mean age of 26.6 ± 4 years. Detailed player and ACL injuryrelated data can be found in Table 1. Moreover, of the total ACL ruptures, 45 (88.2%) were sustained during football matches (friendly and competitive matches) and 6 (11.8%) were sustained during training. The mean time to RTP after a primary ACL rupture was 235 ± 70 days.

**TABLE 1:** Descriptors of players and primary ACL injury-related data (n = 51)^a^.

Age, mean ± *SD*; mean (95%CI) years	26.6 ± 4; 26.6 (25.5–27.7)
Height, mean ± *SD;* mean (95%CI) cm	178.8 ± 6.6; 178.8 (176.9–180.6)
Body mass, mean ± *SD;* mean (95%CI) kg	72.3 ± 5.8; 72.3 (70.7–73.9)
BMI, mean ± *SD;* mean (95%CI) *kg/m*^2^	22.6 ± 1.5;22.6 (22.2–23)
Side of the injury, n (%)	Right, 31 (60.8); Left, 20 (39.2)
Injury on the dominant leg, n (%)	26 (51.0)
Injury on the non-dominant leg, n (%)	25 (49.0)

**Injury mechanism, n (%)**	
Non-contact	24 (47)
Indirect contact	20 (39.2)
Direct contact	7 (13.7)

**Field position, n (%)**	
Defender	21 (41.2)
Midfielder	13 (25.5)
Forward	17 (33.3)

**Age group categories, n (%)**	
≤ 25 years	24 (47.1)
> 25 years	27 (52.9)

**Age by group, *mean ± SD*; mean (95%CI) years**	
Defender	28.1 ± 3.9; 28.1 (26.5–29.8)
Midfielder	25.7 ± 4.3; 25.7 (23.4–28)
Forward	25.2 ± 4.2; 25.2 (23.8–26.8)
≤ 25 years	23 ± 1.7; 23 (22.3–23.7)
> 25 years	29.8 ± 2.2; 29.8 (28.9–30.6)

**Injury context, n (%)**	
Friendly match	3 (5.9)
Competitive match	42 (82.3)
Training	6 (11.8)

Duration of the RTP process, *mean ± SD;* mean (95%CI) days	235.9 ± 71.5; 235.9 (216.3–255.6)

a All data are complete; no missing values were present. Abbreviations: *SD*: Standard deviation; CI: Confidence interval; BMI: Body mass index; RTP: Return to play.

### Level of competition after the RTP (n = 51)

**FIG. 1:**
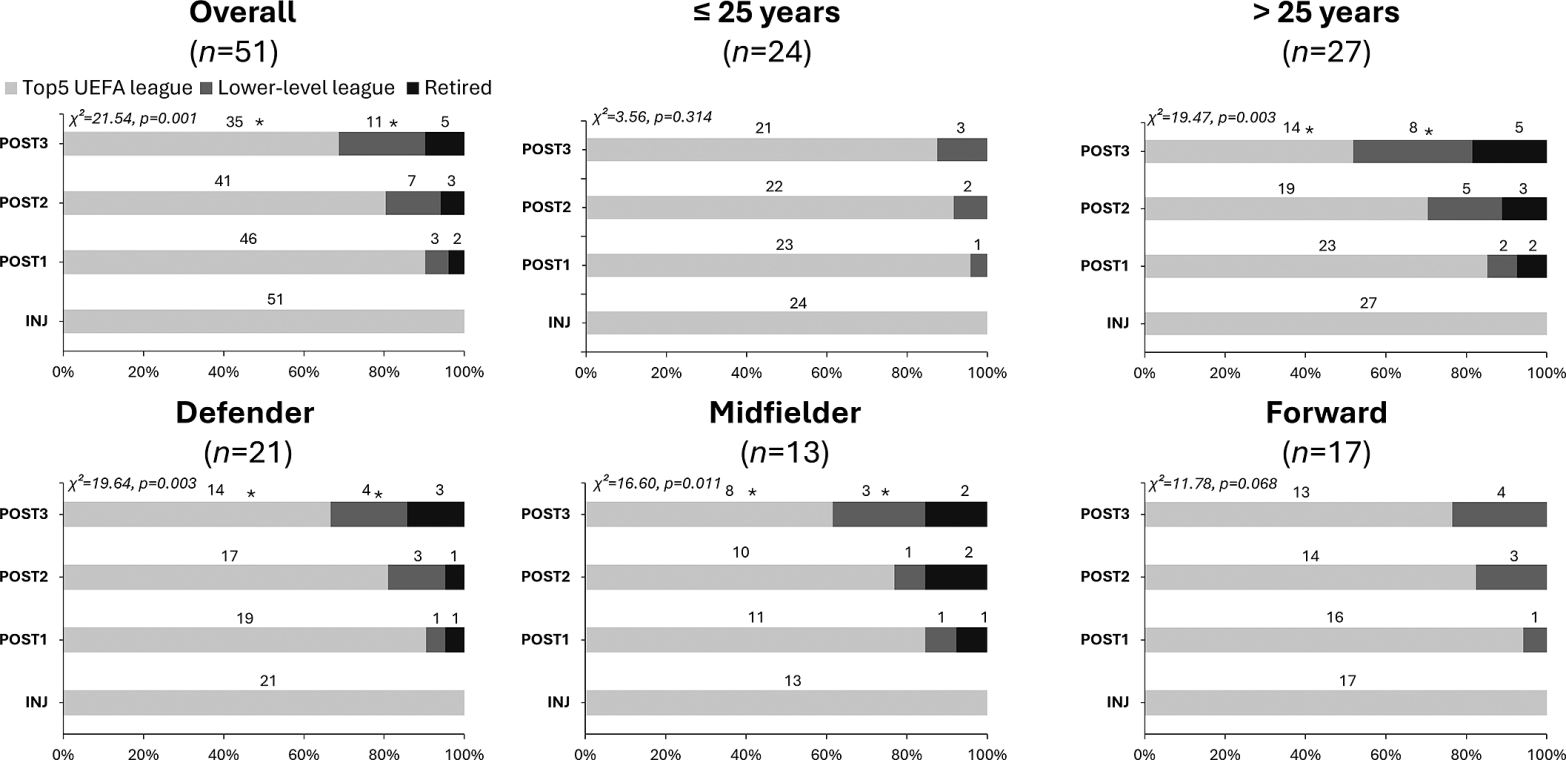
Players’ level of competition during the season of the injury (INJ) and up to three seasons (POST1, POST2, POST3, respectively) after RTP in professional footballers with a primary anterior cruciate ligament rupture. Data are presented overall, depending on the player’s field position and depending on the player’s age at the moment of the injury. All players were playing in LaLiga at the moment of the injury. “Top 5 UEFA league” refers to players who returned to play in teams of the most competitive national leagues in Europe in the different periods of analysis: the English Premier League (England), LaLiga (Spain), Bundesliga (Germany), Serie A (Italy), and Ligue 1 (France). “Lower-level league” refers to players who returned to play in teams of national leagues different from the Top5 leagues in Europe or national leagues outside of Europe. “Retired” relates to players who finished their professional career in different periods of analysis. Statistical analysis compared the distribution of players among the Top 5 UEFA leagues, the Lower-level leagues group and the Retired group. * Indicates that the distribution for a given group was statistically significantly different compared to the INJ season (Chi-square test), based on adjusted standardised residuals exceeding |1.96| (p<0.050).

The level of competition after the RTP of a primary ACL rupture for overall players and stratified by age and playing position is shown in Figure 1. Specifically, 46 (90.2%) football players with a primary ACL rupture went back to play at the same level (top 5 UEFA leagues) during POST1 after the ACL injury, 41 (80.4%) and maintained that level during POST2 and 35 (68.7%) during POST3 (χ^2^ = 21.54, p = 0.001). Out of the 35 players who continued playing at the top level three years after RTP, 34 were still competing in *LaLiga* and 1 in another top-five UEFA league (Ligue 1 [France]). On the other hand, 11 football players moved to a lower-level league at some moment of the follow-up. Particularly, 3 players (5.9%) transferred to a lower-level league during POST1, 4 more players moved to a lower-level league during POST2 (making a total of 13.7%), and 4 more transferred during POST3 (for a total of 21.5%). Related to players who finally ended their careers, a total of 5 players retired over the following three seasons after the injury. Specifically, 2 players (3.9%) retired during POST1, 1 more player retired during POST2 (making a total of 5.9%), and 1 more retired at POST3 (for a total of 9.8%). Overall, the proportion of players in the top 5 UEFA leagues at POST3 was significantly lower compared with INJ (-31.4%, p < 0.05), while the proportion of players in lower leagues and those who had retired at POST3 was significantly higher compared with INJ (21.1%, p < 0.05).

Related to field positional analysis, 19 (90.5%) defenders remained at the same competitive level (first team of the top 5 UEFA leagues) during POST1, 17 (80.9%) during POST2 and 14 (66.7%) at POST3 (χ^2^ = 19.64, p = 0.003). Conversely, 1 (4.8%) moved to a lower league during POST1, 3 (14.3%) at POST2 and 4 (19%) during POST3.

Similar significant findings were observed among midfielders as 11 players (84.6%) returned at the same level at POST1, 10 (76.9%) during POST2 and 8 (61.5%) during POST3 (χ^2^ = 16.60, p = 0.011). Furthermore, 1 player (7.7%) moved to a lower league during POST1 and POST2, increasing to 3 players (23.1%) at POST3. As for retirement, 1 (7.7%) ended his career at POST1, and 2 retired (15.4%) during both POST2 and POST3.

Lastly, among forwards, 16 players (94.1%) returned to the same level during POST1, 14 (82.3%) during POST2 (χ^2^ = 11.78, p = 0.068) and 13 (76.5%) at POST3. In contrast, 1 player (5.9%) moved to a lower league at POST1, 3 did so (17.6%) during POST2 and 4 (23.5%) during POST3.

Regarding age-group analysis, 23 (95%) players aged ≤ 25 years maintained the same competitive level during POST1, 22 (92%) during POST2 and 21 (88%) at POST3 (χ^2^ = 3.56, p = 0.314). In addition, no football player ≤ 25 retired during the three seasons after an ACL rupture. However, the number of players > 25 years old that remained in the same category decreased significantly. Interestingly, 23 (85.2%) players remained at the top 5 UEFA leagues during POST1, 19 (70.4%) at POST2 and 14 (51.9%) at POST3 (χ^2^ = 19.47, p = 0.003). Finally, 2 players (7.4%) ended their career at POST1, 3 (11.1%) at POST2 and 5 (18.5%) at POST3.

### Match running performance metrics after the RTP (n = 34)

**FIG. 2:**
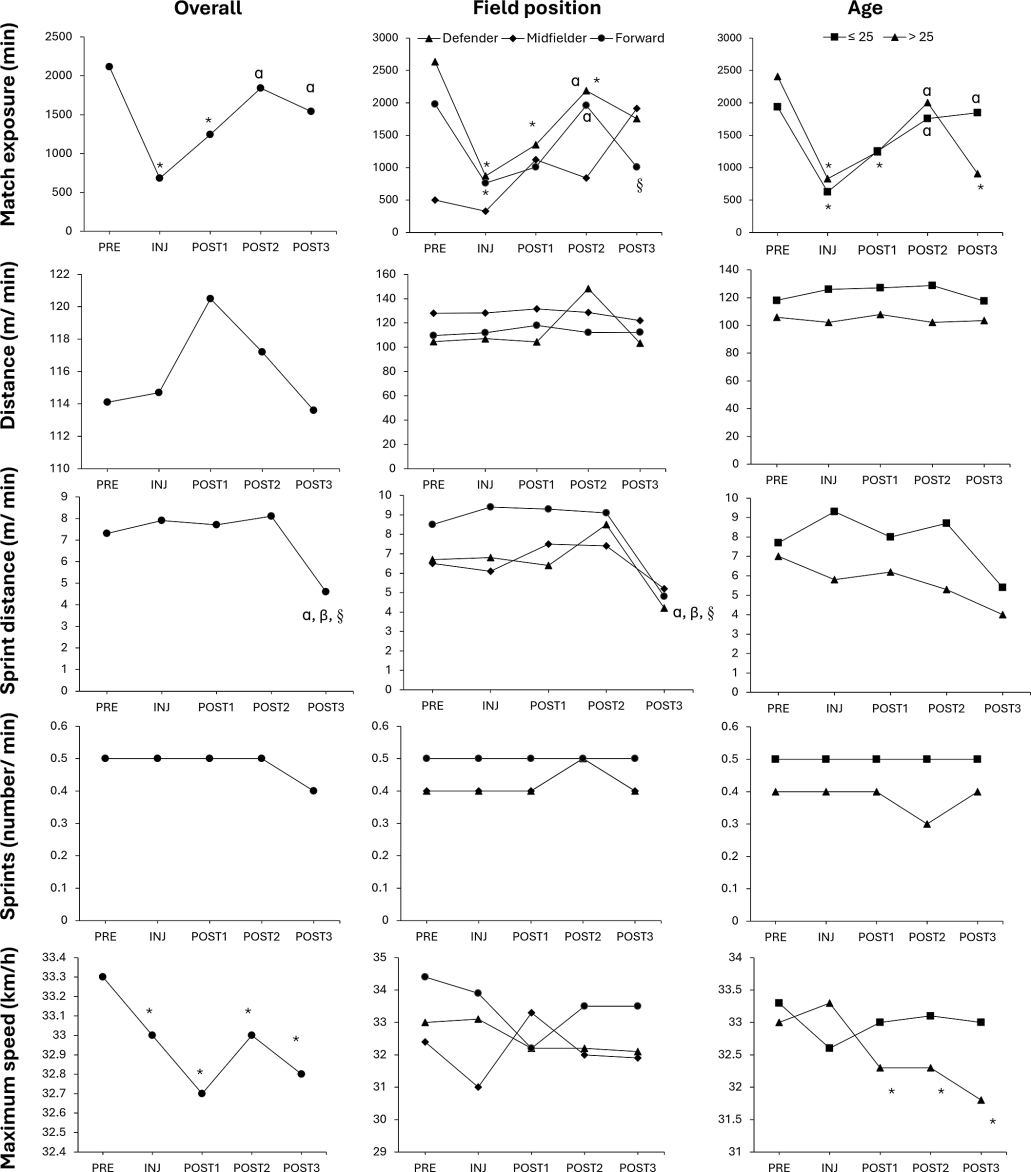
Match data before (PRE), during the season of the injury (INJ), and up to three seasons after the return to play (POST1, POST2, POST3, respectively) in professional football players with a primary anterior cruciate ligament rupture. Data are presented overall (first column), by field position (second column), and by age at the time of injury (third column). Statistical differences were assessed using Friedman tests followed, where significant, by Wilcoxon signed-rank pairwise comparisons with Bonferroni correction. Symbols indicate significant differences (p < 0.050, adjusted) between the following time points: * vs. PRE; α vs. INJ; β vs. POST1; § vs. POST2.

Performance data from the PRE, INJ, POST1, POST2 and POST3 is reported in Figure 2. Players experienced a significant decrease in minutes played during INJ (-67.7%, p < 0.001) in comparison with PRE, and there was a significant increase in match exposure during POST2 (69.0%, p = 0.001) and POST3 (25.2%, p = 0.043) compared to INJ. Regarding running performance, the analysis of sprint distance/min showed differences (Friedman statistic = 13.27, p = 0.010), but no pairwise comparison differences were presented when the Bonferroni correction was applied. However, the maximum running speed during POST1 (-1.9%, p = 0.033) and POST2 (-0.9%, p = 0.020) was significantly lower compared with PRE. Detailed analysis is shown in Supplemental File 1.

Data of performance metrics according to field position are also shown in Figure 2. Regarding field positions, defenders experienced a significant reduction of minutes played during INJ (-66.9%, p = 0.017) and POST1 (-48.7%, p = 0.031) in comparison to PRE. Forwards showed a significant decrease in minutes played during INJ (-61.5%, p = 0.005) compared to PRE, while midfielders did not show significant differences during the seasons after an ACL rupture. For data regarding match running performance metrics, the analysis of sprint distance/min in forwards presented differences (Friedman statistic = 12.47, p = 0.014), but no changes were presented during pairwise comparisons when Bonferroni correction was applied.

Minutes played decreased during INJ compared to PRE in players ≤ 25 years (-67.7%, p = 0.032) and > 25 years (-65.5%, p = 0.004). Only professional football players > 25 years showed a statistically significant drop in their maximum running speed during POST2 (-2.1%, p = 0.017) and POST3 (-3.6%, p = 0.009) when compared to PRE. No significant changes in any performance metrics were reported in professional football players ≤ 25 years. Detailed information about the analysis is shown in Supplemental Files 1 and 2.

## DISCUSSION

This study aimed to examine the impact of an ACL rupture on the level of competition and match running performance metrics over the three postoperative seasons following RTP among professional male football players from *LaLiga*. Using data from the whole sample of players (n = 51) the main outcome was that only about twothirds of the players remained at the top competition level (top 5 UEFA leagues) during POST3 after RTP, while those players aged > 25 were more likely to experience a career decline or to retire. Additionally, analysis of the subset of players who continued playing in *LaLiga* for up to three seasons after their RTP (n = 34) revealed that maximum running speed decreased during POST1 and POST2 (compared with PRE). Only those players > 25 years old experienced a significant decrease in the maximum speed in POST2 and POST3. These findings partially support our initial hypothesis, indicating a sustained reduction in maximum running speed for up to three seasons after RTP, with older players being more susceptible to performance decline. However, no significant differences were found in the remaining running performance variables. These outcomes highlight the longlasting negative impact of an ACL rupture on both career sustainability and running match performance (i.e., maximum speed), therefore highlighting the importance of targeted rehabilitation and performance monitoring after ACL injuries, especially for older players.

Concerning the level of competition of *LaLiga* players, the present study showed that only 68.6% of them remained at the same elite level of competition, while 9.8% retired within three seasons after their RTP following an ACL injury. Interestingly, our data showed that players who finally retired were all > 25 years at the moment of the injury and mainly affected defenders and midfielders. Although playing styles and game demands may vary across regions, the findings of the current study are in line with previous research reporting an important reduction in the number of players (50–65%) remaining at the same level of competition [4, 14] and retired (12–13%) [14, 20] after an ACL rupture and within three years of RTP in other European Leagues. The top 5 UEFA elite leagues (e.g., *LaLiga* [Spain], Serie A [Italy], Bundesliga [Germany], Premier League [England] and Ligue 1 [France]) are considered the most demanding leagues in the world (and specifically *LaLiga* in Spain) [35], which reinforces the idea that players need to be in optimal physical condition in a short period of time. Therefore, the finding that 31.4% of players in the present study were unable to remain in any of these top-tier leagues is likely related to the high physical demands associated with elite-level football. On the other hand, the reason why players retired, specifically defenders and midfielders, could be due to the higher age of these players in our sample, a decrease in their physical condition [36], the fear of reinjury and/or the pain or instability in the injured knee [37]. Regarding the minutes played, ACL-injured players exhibit reduced participation with the team, similar to previous research that reported prolonged participation deficits during POST1 and POST2 [14, 16, 17, 19]. This reduction in playing time may be attributed to a decline in physical fitness resulting from the prolonged rehabilitation process and the gradual reintegration into competitive play [38]. Additionally, it may reflect a cautious approach by coaching and medical staff to protect players from reinjury following an ACL reconstruction [7].

Although the monitoring of running performance metrics is considered essential to examine the rehabilitation process and make clinical decisions for RTP, little is known about how ACL injury affects the match running performance metrics after effective RTP [22, 23]. In this context, it is difficult to make comparisons with the literature as no previous study has reported running performance metrics in the seasons after RTP following an ACL rupture in football players. Additionally, in the present study, the running performance analysis was limited to players who remained in *LaLiga* for up to three years after return to play, which may represent a potential selection bias, as only the performance metrics of players capable of competing at a level sufficient to remain in LaLiga teams were analysed. This implies that the main findings related to running performance metrics should be interpreted as a conservative estimate for the entire cohort of ACL-injured players. Still, our data demonstrate a long-lasting negative impact on running match performance and career sustainability, particularly as players tend to be used less by coaches and exhibit reduced maximum speed during the years following an ACL injury. A potential explanation of the results for this reduction could be related to the persistent deficits in strength and power observed during the final phase of the ACL rehabilitation process in previous studies involving football players [39, 40]. As a contributing factor, reduced strength and power have been associated with lower levels of sprint performance [41, 42] as reflected in the lower maximum speed of each season after an ACL injury. Therefore, rehabilitation protocols should continue to emphasise explosive strength and power development well after the initial RTP to mitigate these long-term speed deficits.

In addition, the results showed that the maximum speed of the season decreases mainly in players > 25 years (Figure 2). Linked with our findings, previous research also reported that it took longer for players older than 25 years to regain their pre-injury performance level compared with younger players [14]. These data indicate that the older players could have more problems recovering their baseline running performance (i.e., maximum speed) values after an ACL injury. Previous findings [43, 44] observed that the peak of performance in footballers was between 25–27 years, so from this age on, it is likely that match performance starts to decline. The findings of this study suggest that in players with an ACL injury, the typical agerelated performance decline may be exacerbated by the injury itself, highlighting the need for targeted rehabilitation strategies. Regaining running performance appears to be a key factor in ensuring career longevity for these players. Also, over the years (particularly > 30 years), players begin to decrease their running performance values, especially the total distance covered and number of sprints [45]. This contrasts with our findings, in which only maximum speed was affected throughout the season, particularly in players > 25 years. Therefore, these results could be relevant in the process of RTP, so those match running performance metrics that players have more difficulty recovering from after an ACL injury should be trained and improved to bring the player back to the highest level of performance, especially in players > 25 years. Based on these results, the significant reduction in maximum seasonal speed observed after an ACL rupture underscores the importance of implementing reconditioning strategies that specifically target the recovery of maximum speed after RTP. Overall, these findings highlight the need to restore pre-injury neuromuscular function during rehabilitation, reconditioning, and the RTP process [9, 23]. However, more research is needed to identify the possible influence of concomitant knee injuries on the recovery trajectories and running match performance after RTP.

This observational study has notable strengths. Primarily, it provides an exceptional access to longitudinal data on elite-level footballers in *LaLiga*, offering valuable insights into a population that is typically difficult to reach for research purposes. Importantly, the data were obtained directly from *LaLiga* through the Mediacoach^®^ system, a video-tracking system that provides consistent data on key external load variables. Nevertheless, the broader challenge of harmonising external load quantification across different tracking technologies remains an important methodological issue in sports science research [46]. To the best of our knowledge, this is the first study to assess match running performance metrics in professional football players over three seasons after an ACL surgery in *LaLiga*, although the analysis was limited to the subset of participants who continued competing in this league after RTP. Furthermore, analyses were stratified by playing position and age group, ensuring that the findings are practically relevant for medical and technical staff. Hence, several limitations must be considered when interpreting the results of this study. First, we were unable to specify the characteristics of the rehabilitation process followed by each player, which could be an important factor in monitoring running performance recovery, particularly for players who were not immediately selected to participate in matches. Second, ACL ruptures were defined irrespective of concomitant injuries. While this reflects the clinical reality of football-related ACL tears, it may introduce heterogeneity in recovery trajectories and performance outcomes. Future studies should consider profiling isolated and associated injuries separately, in line with methodological standards proposed for injury profiling studies [47, 48]. Third, the sample size, particularly when stratified by age and field position, was relatively small, potentially limiting the statistical power and precluding a detailed analysis by age and playing position. Additionally, match running performance was analysed only in those who remained in *LaLiga* after RTP (34 players out of the total number of 51 football players), meaning the findings may not fully capture the outcomes of players who moved to other leagues or levels of competition. Restricting the match running performance analysis to players who remained in *LaLiga* introduced a potential selection bias, as it is likely that those with less severe performance declines were more able to continue competing at this level; consequently, the true impact of an ACL rupture on performance may have been underestimated by our methodological approach. It is also important to note that the match running performance analysis was limited to elite-level male football players. Therefore, the results should not be extrapolated to other populations, such as semi-professional or female football players. In addition, it should be acknowledged that playing position may influence running performance metrics and players’ adaptations before and after an ACL injury, although such effects have been reported in elite female footballers [49]. Lastly, this study did not include a control group of non-injured players, as it was challenging to identify healthy individuals in *LaLiga* with characteristics, field position, and age. This limitation constrains the ability to determine whether the observed reductions are specific to an ACL injury. However, it is important to note that uninjured ACL players may also experience extended periods of inactivity due to other injuries or contextual factors, which makes the inclusion of the control group less reliable. Future research should address these gaps by including broader performance indicators, larger and more diverse samples and by examining both training and match performance across different competitive levels.

## CONCLUSIONS

In summary, a notable portion of professional male football players from *LaLiga* who suffered a complete ACL rupture experienced a decrease in both their level of competition (moving down to lower-level teams) and running performance parameters (specifically their maximum running speed) for at least three seasons following their RTP. Moreover, players often fail to regain pre-injury match exposure until beyond the POST2. Additionally, players > 25 years presented greater running performance reductions than younger players. Given the persistent decline in the maximum speed observed up to three seasons after an ACL surgery, reconditioning programs should prioritise restoring high-intensity running and sprinting capacity. Special attention should be given to players > 25 years old, who appear to experience greater and more prolonged performance deficits. Additionally, clubs and practitioners should be aware that players may require up to three years to regain their pre-injury match playing time; however, some may never fully recover their pre-injury maximum speed, even three years after returning to play.

## Supplementary Material

Match running performance upon return to play in professional male LaLiga football players following anterior cruciate ligament rupture

## References

[1] Requejo-Herrero P, Pineda-Galan C, Medina-Porqueres I. Anterior cruciate ligament ruptures in Spanish soccer first division: An epidemiological retrospective study. Knee. 2023; 41:48–57. 36630867 10.1016/j.knee.2022.11.014

[2] Erickson BJ, Harris JD, Cvetanovich GL, et al. Performance and Return to Sport After Anterior Cruciate Ligament Reconstruction in Male Major League Soccer Players. Orthop J Sports Med. 2013; 1(2):1–7. 10.1177/2325967113497189 PMC4555483 26535238

[3] Dhahbi W, Materne O, Chamari K. Rethinking knee injury prevention strategies: joint-by-joint training approach paradigm versus traditional focused knee strengthening. Biol Sport. 2025; 42(4):59–65. 10.5114/biolsport.2025.148544 PMC12490306 41048234

[4] Waldén M, Hägglund M, Magnusson H, Ekstrand J. ACL injuries in men’s professional football: a 15-year prospective study on time trends and return-to-play rates reveals only 65% of players still play at the top level 3 years after ACL rupture. Br J Sports Med. 2016; 50(12):744–750. 27034129 10.1136/bjsports-2015-095952

[5] Krutsch W, Memmel C, Alt V, et al. Timing return-to-competition: a prospective registration of 45 different types of severe injuries in Germany’s highest football league. Arch Orthop Trauma Surg. 2022; 142(3):455–463. 33779832 10.1007/s00402-021-03854-8 PMC8843858

[6] Molloy MG, Molloy CB. Contact sport and osteoarthritis. Br J Sports Med. 2011; 45(4):275–277. 21444375 10.1136/bjsm.2011.083956

[7] Della Villa F, Hägglund M, Della Villa S, Ekstrand J, Waldén M. High rate of second ACL injury following ACL reconstruction in male professional footballers: an updated longitudinal analysis from 118 players in the UEFA Elite Club Injury Study. Br J Sports Med. 2021; 55(23):1350–1356. 33846157 10.1136/bjsports-2020-103555 PMC8606446

[8] Mujika I, Padilla S. Detraining: loss of training-induced physiological and performance adaptations. Part I: short term insufficient training stimulus. Sports Med. 2000; 30(2):79–87. 10966148 10.2165/00007256-200030020-00002

[9] Buckthorpe M, Frizziero A, Roi GS. Update on functional recovery process for the injured athlete: return to sport continuum redefined. Br J Sports Med. 2019; 53(5):265–267. 30269097 10.1136/bjsports-2018-099341

[10] Requena B, García I, Suárez-Arrones L, Sáez De Villarreal E, Naranjo Orellana J, Santalla A. Off-Season Effects on Functional Performance, Body Composition, and Blood Parameters in Top-Level Professional Soccer Players. J Strength Cond Res. 2017; 31(4):939–946. 27438062 10.1519/JSC.0000000000001568

[11] Koundourakis NE, Androulakis NE, Malliaraki N, Tsatsanis C, Venihaki M, Margioris AN. Discrepancy between exercise performance, body composition, and sex steroid response after a six-week detraining period in professional soccer players. PLoS One. 2014; 9(2):e87803. 24586293 10.1371/journal.pone.0087803 PMC3929557

[12] Suarez-Arrones L, Lara-Lopez P, Maldonado R, et al. The effects of detraining and retraining periods on fat-mass and fat-free mass in elite male soccer players. PeerJ. 2019; 7:e7466. 31423358 10.7717/peerj.7466 PMC6697042

[13] Alashram AR, D’Onofrio R, Sannicandro I, et al. Return to training in soccer players after anterior cruciate ligament reconstruction: reflections and considerations. J Sports Med Phys Fitness. 2024; 64(6):578–587. 38324269 10.23736/S0022-4707.23.15448-X

[14] Mazza D, Viglietta E, Monaco E, et al. Impact of Anterior Cruciate Ligament Injury on European Professional Soccer Players. Orthop J Sports Med. 2022; 10(2):23259671221076864. 10.1177/23259671221076865 PMC8873562 35224121

[15] Zaffagnini S, Grassi A, Muccioli GMM, et al. Return to sport after anterior cruciate ligament reconstruction in professional soccer players. Knee. 2014; 21(3):731–735. 24593869 10.1016/j.knee.2014.02.005

[16] Niederer D, Engeroff T, Wilke J, Vogt L, Banzer W. Return to play, performance, and career duration after anterior cruciate ligament rupture: A case-control study in the five biggest football nations in Europe. Scand J Med Sci Sports. 2018; 28(10):2226–2233. 29927499 10.1111/sms.13245

[17] Barth KA, Lawton CD, Touhey DC, et al. The negative impact of anterior cruciate ligament reconstruction in professional male footballers. Knee. 2019; 26(1):142–148. 30449615 10.1016/j.knee.2018.10.004

[18] Balendra G, Jones M, Borque KA, Willinger L, Pinheiro VH, Williams A. Factors affecting return to play and graft re-rupture after primary ACL reconstruction in professional footballers. Knee Surg Sports Traumatol Arthrosc. 2022; 30(7):2200–2208. doi: 10.1007/S00167-021-06765-8. 34636948

[19] Forsythe B, Lavoie-Gagne OZ, Forlenza EM, Diaz CC, Mascarenhas R. Return-to-Play Times and Player Performance After ACL Reconstruction in Elite UEFA Professional Soccer Players: A Matched-Cohort Analysis From 1999 to 2019. Orthop J Sports Med. 2021; 9(5):23259671211008892. 34104662 10.1177/23259671211008892 PMC8165856

[20] Farinelli L, Abermann E, Meena A, Ueblacker P, Hahne J, Fink C. Return to Play and Pattern of Injury After ACL Rupture in a Consecutive Series of Elite UEFA Soccer Players. Orthop J Sports Med. 2023; 11(3):23259671231153628. 10.1177/23259671231153629 PMC9989402 36896098

[21] Manojlovic M, Ninkovic S, Matic R, et al. Return to Play and Performance After Anterior Cruciate Ligament Reconstruction in Soccer Players: A Systematic Review of Recent Evidence. Sports Med. 2024; 54(8):2097–2108. 38710914 10.1007/s40279-024-02035-y PMC11329701

[22] Taberner M, Van Dyk N, Allen T, et al. Physical preparation and return to performance of an elite female football player following ACL reconstruction: A journey to the FIFA Women’s World Cup. BMJ Open Sport Exerc Med. 2020; 6(1):e000843. 10.1136/bmjsem-2020-000843 PMC8323467 34422284

[23] Picinini F, Della Villa F, Tallent J, et al. High Return to Competition Rate After On-Field Rehabilitation in Competitive Male Soccer Players After ACL Reconstruction: GPS Tracking in 100 Consecutive Cases. Orthop J Sports Med. 2025; 13(3):23259671251320092. 10.1177/23259671251320093 PMC11881939 40052178

[24] Di Salvo V, Benito P, Calderón F, Di Salvo M, Pigozzi F. Activity profile of elite goalkeepers during football match-play. J Sports Med Phys Fitness. 2008; 48(4):443–446. 18997646

[25] Perez-Arroniz M, Calleja-González J, Zabala-Lili J, Zubillaga A. The soccer goalkeeper profile: bibliographic review. Phys Sportsmed. 2023; 51(3):193–202. 35157536 10.1080/00913847.2022.2040889

[26] White A, Hills SP, Cooke CB, et al. Match-Play and Performance Test Responses of Soccer Goalkeepers: A Review of Current Literature. Sports Med. 2018; 48(11):2497–2516. 30144021 10.1007/s40279-018-0977-2

[27] Waldén M, Hägglund M, Magnusson H, Ekstrand J. Anterior cruciate ligament injury in elite football: a prospective three-cohort study. Knee Surg Sports Traumatol Arthrosc. 2011; 19(1):11–19. 20532869 10.1007/s00167-010-1170-9

[28] Moreno-Perez V, Campos-Vazquez MA, Toscano J, et al. Influence of the Weekly and Match-play Load on Muscle Injury in Professional Football Players. Int J Sports Med. 2022; 43(9):783–790. 35189659 10.1055/a-1533-2110

[29] Pons E, García-Calvo T, Resta R, et al. A comparison of a GPS device and a multi-camera video technology during official soccer matches: Agreement between systems. PLoS One. 2019; 14(8). 10.1371/journal.pone.0220729 PMC6687125 31393932

[30] Felipe JL, Garcia-Unanue J, Viejo-Romero D, Navandar A, Sánchez-Sánchez J. Validation of a Video-Based Performance Analysis System (Mediacoach^®^) to Analyze the Physical Demands during Matches in LaLiga. Sensors. 2019; 19(19):4113. 31547591 10.3390/s19194113 PMC6806213

[31] Leventer L, Eek F, Hofstetter S, Lames M. Injury Patterns among Elite Football Players: A Media-based Analysis over 6 Seasons with Emphasis on Playing Position. Int J Sports Med. 2016; 37(11):898–908. 27467906 10.1055/s-0042-108201

[32] Harris CR, Millman KJ, van der Walt SJ, et al. Array programming with NumPy. Nature. 2020; 585(7825):357–362. 32939066 10.1038/s41586-020-2649-2 PMC7759461

[33] Virtanen P, Gommers R, Oliphant TE, et al. SciPy 1.0: fundamental algorithms for scientific computing in Python. Nat Methods. 2020; 17(3):261–272. 32015543 10.1038/s41592-019-0686-2 PMC7056644

[34] Vallat R. Pingouin: statistics in Python. J Open Source Softw. 2018; 3(31):1026.

[35] Barba GJM, Luján JCB, Enriquez SIR, Gómez ORO, Sambrano GS, Delgado JCG. Análisis comparativo de estructuras competitivas y aspectos sistemáticos en las mejores ligas de futbol mundial. Rev Mex Cienc Cult Fis. 2024; 3(9):32–46.

[36] Britt E, Ouillette R, Edmonds E, et al. The Challenges of Treating Female Soccer Players With ACL Injuries: Hamstring Versus Bone-Patellar Tendon-Bone Autograft. Orthop J Sports Med. 2020; 8(11). 10.1177/2325967120964884 PMC7708716 33294473

[37] Sandon A, Engström B, Forssblad M. High Risk of Further Anterior Cruciate Ligament Injury in a 10-Year Follow-up Study of Anterior Cruciate Ligament-Reconstructed Soccer Players in the Swedish National Knee Ligament Registry. Arthroscopy. 2020; 36(1):189–195. 31439457 10.1016/j.arthro.2019.05.052

[38] Almeida AM, Silva PRS, Pedrinelli A, Hernandez AJ. Aerobic fitness in professional soccer players after anterior cruciate ligament reconstruction. PLoS One. 2018; 13(3):e0194432. 29566090 10.1371/journal.pone.0194432 PMC5864031

[39] Maestroni L, Turner A, Papadopoulos K, et al. Comparison of Strength and Power Characteristics Before ACL Rupture and at the End of Rehabilitation Before Return to Sport in Professional Soccer Players. Sports Health. 2023; 15(6):814–823. 37203795 10.1177/19417381231171566 PMC10606975

[40] Timmins RG, Bourne MN, Shield AJ, Williams MD, Lorenzen C, Opar DA. Biceps Femoris Architecture and Strength in Athletes with a Previous Anterior Cruciate Ligament Reconstruction. Med Sci Sports Exerc. 2016; 48(3):337–345. 26429732 10.1249/MSS.0000000000000783

[41] Belli A, Kyröläinen H, Komi PV. Moment and power of lower limb joints in running. Int J Sports Med. 2002; 23(2):136–141. 11842362 10.1055/s-2002-20136

[42] Mann RA, Hagy J. Biomechanics of walking, running, and sprinting. Am J Sports Med. 1980; 8(5):345–350. 7416353 10.1177/036354658000800510

[43] Dendir S. When do soccer players peak? A note. J Sports Anal. 2016; 2(2):89–105.

[44] Rey E, Costa PB, Corredoira FJ, Sal De Rellán Guerra A. Effects of Age on Physical Match Performance in Professional Soccer Players. J Strength Cond Res. 2023; 37(6):1244–1249. 31268996 10.1519/JSC.0000000000003244

[45] Sal de Rellán-Guerra A, Rey E, Kalén A, Lago-Peñas C. Age-related physical and technical match performance changes in elite soccer players. Scand J Med Sci Sports. 2019; 29(9):1421–1427. 31099117 10.1111/sms.13463

[46] Dhahbi W, Chaabene H, Pyne DB, Chamari K. Standardizing the Quantification of External Load Across Different Training Modalities: A Critical Need in Sport-Science Research. Int J Sports Physiol Perform. 2024; 19(11):1173–1175. 39326859 10.1123/ijspp.2024-0366

[47] Dhahbi W, Ben Saad H, Dergaa I, Souaifi M, Chamari K. Injury Profiling in Male Police Cadets During Initial Training Phase: A Retrospective Cohort Study. Am J Mens Health. 2024; 18(6). 10.1177/15579883241304584 PMC11626666 39651577

[48] Bahr R, Clarsen B, Derman W, et al. International Olympic Committee Consensus Statement: Methods for Recording and Reporting of Epidemiological Data on Injury and Illness in Sports 2020 (Including the STROBE Extension for Sports Injury and Illness Surveillance (STROBE-SIIS)). Orthop J Sports Med. 2020; 8(2). 10.1177/2325967120902908 PMC7029549 32118084

[49] Slimani M, Ghouili H, Dhahbi W, et al. Position-specific biomarker responses to match vs. VAMEVAL test modalities in elite female soccer players: a comparative analysis study. Cogent Soc Sci. 2025; 11(1).

