# Supplementary material for: Match running performance upon return to play in professional male LaLiga football players following anterior cruciate ligament rupture
Source: Biol Sport. 2026 Apr 13;43:1161–70. doi: 10.5114/biolsport.2026.160857 (PMC13343279; doi:10.5114/biolsport.2026.160857)
Supplement: Match running performance upon return to play in professional male LaLiga football players following anterior cruciate ligament rupture [file JBS-43-57884-s1.pdf]

SUPPLEMENTARY MATERIALS

SUPPLEMENTAL FILE 1. Match data before (PRE), the season of the injury (INJ) and up to three seasons (POST1, POST2, POST3, respectively) after the RTP in professional football players with primary anterior cruciate ligament injury for overall and depending on their age (n=34).

Variables	Field position	Friedman Statistic	p	Season 1	Season 2	Median1 (IQR 1)	Median 2 (IQR 2)	Wilcoxon Statistic	p adjusted	95%CI
Minutes of match play (min)	OVERALL	29.11	**< 0.001**	PRE	INJ	2115.94 (1502.13)	684.11 (1050.87)	50	**< 0.001**	463.61; 1499.13
				PRE	POST1	2115.94 (1502.13)	1242.35 (1310.44)	157	0.153	17.02; 1392.47
				PRE	POST2	2115.94 (1502.13)	1839.84 (1445.84)	195	0.811	52.23; 526.17
				PRE	POST3	2115.94 (1502.13)	1540.46 (1322.13)	195	0.811	19.81; 1227.67
				INJ	POST1	684.11 (1050.87)	1242.35 (1310.44)	199	0.941	-1236.28; 134.22
				INJ	POST2	684.11 (1050.87)	1839.84 (1445.84)	81	**0.001**	-1291.47; -252.33
				INJ	POST3	684.11 (1050.87)	1540.46 (1322.13)	134	**0.043**	-1113.42; -112.71
				POST1	POST2	1242.35 (1310.44)	1839.84 (1445.84)	206	1.000	-990.5; 18.7
				POST1	POST3	1242.35 (1310.44)	1540.46 (1322.13)	224	1.000	-656.9; 137.51
				POST2	POST3	1839.84 (1445.84)	1540.46 (1322.13)	257	1.000	-149.13; 341.76
	≤ 25 YEARS	16.47	**0.002**	PRE	INJ	1938.95 (1688.39)	626.21 (802.51)	29	**0.032**	361.71; 1546.38
				PRE	POST1	1938.95 (1688.39)	1257.16 (1230.44)	78	1.000	-237.58; 917.28
				PRE	POST2	1938.95 (1688.39)	1755.93 (1365.64)	80	1.000	-174.26; 599.25
				PRE	POST3	1938.95 (1688.39)	1846.03 (1342.49)	100	1.000	-638.14; 852.83
				INJ	POST1	626.21 (802.51)	1257.16 (1230.44)	56	0.696	-1387.46; 159.49
				INJ	POST2	626.21 (802.51)	1755.93 (1365.64)	19	**0.006**	-1339.16; -216.23
				INJ	POST3	626.21 (802.51)	1846.03 (1342.49)	22	**0.010**	-1568.91; -156.59
				POST1	POST2	1257.16 (1230.44)	1755.93 (1365.64)	87	1.000	-990.5; 582.44
				POST1	POST3	1257.16 (1230.44)	1846.03 (1342.49)	65	1.000	-910.39; 170.59
				POST2	POST3	1755.93 (1365.64)	1846.03 (1342.49)	67	1.000	-593.62; 103.77
	> 25 YEARS	17.03	**0.002**	PRE	INJ	2408.4 (1311.7)	830.39 (1253.25)	2	**0.004**	452.04; 2126.19
				PRE	POST1	2408.4 (1311.7)	1242.35 (1473.04)	17	0.245	213.9; 1847.59
				PRE	POST2	2408.4 (1311.7)	2005.74 (1305.38)	26	1.000	-82.14; 1365.3
				PRE	POST3	2408.4 (1311.7)	905.59 (1297.54)	12	0.085	141.13; 1858.36
				INJ	POST1	830.39 (1253.25)	1242.35 (1473.04)	42	1.000	-996.68; 489.64
				INJ	POST2	830.39 (1253.25)	2005.74 (1305.38)	17	0.245	-1535.78; -33
				INJ	POST3	830.39 (1253.25)	905.59 (1297.54)	42	1.000	-1114.58; 638.41
				POST1	POST2	1242.35 (1473.04)	2005.74 (1305.38)	25	0.906	-1229.63; 31.73
				POST1	POST3	1242.35 (1473.04)	905.59 (1297.54)	48	1.000	-656.9; 709.66
				POST2	POST3	2005.74 (1305.38)	905.59 (1297.54)	20	0.419	0.04; 903.94

SUPPLEMENTAL FILE 1. Continue

Variables	Field position	Friedman Statistic	p	Season 1	Season 2	Median1 (IQR 1)	Median 2 (IQR 2)	Wilcoxon Statistic	p adjusted	95%CI
Total distance/min (m/min)	OVERALL	7.39	0.117	PRE	INJ	114.12 (29.37)	114.74 (95.32)			
				PRE	POST1	114.12 (29.37)	120.51 (94.36)			
				PRE	POST2	114.12 (29.37)	117.19 (97.33)			
				PRE	POST3	114.12 (29.37)	113.59 (80.42)			
				INJ	POST1	114.74 (95.32)	120.51 (94.36)			
				INJ	POST2	114.74 (95.32)	117.19 (97.33)			
				INJ	POST3	114.74 (95.32)	113.59 (80.42)			
				POST1	POST2	120.51 (94.36)	117.19 (97.33)			
				POST1	POST3	120.51 (94.36)	113.59 (80.42)			
				POST2	POST3	117.19 (97.33)	113.59 (80.42)			
	≤ 25 YEARS	8.31	0.081	PRE	INJ	118.01 (41.74)	126.01 (91.64)			
				PRE	POST1	118.01 (41.74)	127.05 (109.01)			
				PRE	POST2	118.01 (41.74)	128.69 (93.21)			
				PRE	POST3	118.01 (41.74)	117.63 (93.92)			
				INJ	POST1	126.01 (91.64)	127.05 (109.01)			
				INJ	POST2	126.01 (91.64)	128.69 (93.21)			
				INJ	POST3	126.01 (91.64)	117.63 (93.92)			
				POST1	POST2	127.05 (109.01)	128.69 (93.21)			
				POST1	POST3	127.05 (109.01)	117.63 (93.92)			
				POST2	POST3	128.69 (93.21)	117.63 (93.92)			
	> 25 YEARS	3.89	0.422	PRE	INJ	105.77 (22.24)	102.19 (22.22)			
				PRE	POST1	105.77 (22.24)	107.92 (30.09)			
				PRE	POST2	105.77 (22.24)	102.05 (29.34)			
				PRE	POST3	105.77 (22.24)	103.4 (31.41)			
				INJ	POST1	102.19 (22.22)	107.92 (30.09)			
				INJ	POST2	102.19 (22.22)	102.05 (29.34)			
				INJ	POST3	102.19 (22.22)	103.4 (31.41)			
				POST1	POST2	107.92 (30.09)	102.05 (29.34)			
				POST1	POST3	107.92 (30.09)	103.4 (31.41)			
				POST2	POST3	102.05 (29.34)	103.4 (31.41)			

SUPPLEMENTAL FILE 1. Continue

Variables	Field position	Friedman Statistic	p	Season 1	Season 2	Median1 (IQR 1)	Median 2 (IQR 2)	Wilcoxon Statistic	p adjusted	95%CI
Sprint distance/min (m/min)	OVERALL	13.27	**0.01**	PRE	INJ	7.29 (4.35)	7.92 (4.16)	214	1.000	-1.03; 0.14
				PRE	POST1	7.29 (4.35)	7.71 (5.2)	205	1.000	-1.15; -0.07
				PRE	POST2	7.29 (4.35)	8.14 (5.36)	267	1.000	-0.91; 0.59
				PRE	POST3	7.29 (4.35)	4.62 (6)	195	0.811	-0.05; 3.21
				INJ	POST1	7.92 (4.16)	7.71 (5.2)	271	1.000	-0.95; 0.78
				INJ	POST2	7.92 (4.16)	8.14 (5.36)	268	1.000	-0.68; 0.72
				INJ	POST3	7.92 (4.16)	4.62 (6)	140	0.061	0.49; 3.61
				POST1	POST2	7.71 (5.2)	8.14 (5.36)	248	1.000	-0.48; 1.13
				POST1	POST3	7.71 (5.2)	4.62 (6)	147	0.091	0.6; 3.28
				POST2	POST3	8.14 (5.36)	4.62 (6)	157	0.153	-0.05; 2.87
	≤ 25 YEARS	9.4	0.052	PRE	INJ	7.7 (3.45)	9.25 (4.46)			
				PRE	POST1	7.7 (3.45)	8.02 (6.45)			
				PRE	POST2	7.7 (3.45)	8.73 (2.93)			
				PRE	POST3	7.7 (3.45)	5.42 (7.1)			
				INJ	POST1	9.25 (4.46)	8.02 (6.45)			
				INJ	POST2	9.25 (4.46)	8.73 (2.93)			
				INJ	POST3	9.25 (4.46)	5.42 (7.1)			
				POST1	POST2	8.02 (6.45)	8.73 (2.93)			
				POST1	POST3	8.02 (6.45)	5.42 (7.1)			
				POST2	POST3	8.73 (2.93)	5.42 (7.1)			
	> 25 YEARS	5.77	0.217	PRE	INJ	7.02 (5.82)	5.75 (3.14)			
				PRE	POST1	7.02 (5.82)	6.24 (5.93)			
				PRE	POST2	7.02 (5.82)	5.29 (7.26)			
				PRE	POST3	7.02 (5.82)	4 (4.99)			
				INJ	POST1	5.75 (3.14)	6.24 (5.93)			
				INJ	POST2	5.75 (3.14)	5.29 (7.26)			
				INJ	POST3	5.75 (3.14)	4 (4.99)			
				POST1	POST2	6.24 (5.93)	5.29 (7.26)			
				POST1	POST3	6.24 (5.93)	4 (4.99)			
				POST2	POST3	5.29 (7.26)	4 (4.99)			

SUPPLEMENTAL FILE 1. Continue

Variables	Field position	Friedman Statistic	p	Season 1	Season 2	Median1 (IQR 1)	Median 2 (IQR 2)	Wilcoxon Statistic	p adjusted	95%CI
Sprints/min (n/min)	OVERALL	4.35	0.36	PRE	INJ	0.45 (0.24)	0.46 (0.26)			
				PRE	POST1	0.45 (0.24)	0.48 (0.32)			
				PRE	POST2	0.45 (0.24)	0.51 (0.3)			
				PRE	POST3	0.45 (0.24)	0.42 (0.33)			
				INJ	POST1	0.46 (0.26)	0.48 (0.32)			
				INJ	POST2	0.46 (0.26)	0.51 (0.3)			
				INJ	POST3	0.46 (0.26)	0.42 (0.33)			
				POST1	POST2	0.48 (0.32)	0.51 (0.3)			
				POST1	POST3	0.48 (0.32)	0.42 (0.33)			
				POST2	POST3	0.51 (0.3)	0.42 (0.33)			
	≤ 25 YEARS	3.64	0.457	PRE	INJ	0.46 (0.19)	0.52 (0.22)			
				PRE	POST1	0.46 (0.19)	0.5 (0.37)			
				PRE	POST2	0.46 (0.19)	0.54 (0.23)			
				PRE	POST3	0.46 (0.19)	0.45 (0.34)			
				INJ	POST1	0.52 (0.22)	0.5 (0.37)			
				INJ	POST2	0.52 (0.22)	0.54 (0.23)			
				INJ	POST3	0.52 (0.22)	0.45 (0.34)			
				POST1	POST2	0.5 (0.37)	0.54 (0.23)			
				POST1	POST3	0.5 (0.37)	0.45 (0.34)			
				POST2	POST3	0.54 (0.23)	0.45 (0.34)			
	> 25 YEARS	3.03	0.553	PRE	INJ	0.43 (0.27)	0.36 (0.16)			
				PRE	POST1	0.43 (0.27)	0.36 (0.33)			
				PRE	POST2	0.43 (0.27)	0.33 (0.38)			
				PRE	POST3	0.43 (0.27)	0.4 (0.34)			
				INJ	POST1	0.36 (0.16)	0.36 (0.33)			
				INJ	POST2	0.36 (0.16)	0.33 (0.38)			
				INJ	POST3	0.36 (0.16)	0.4 (0.34)			
				POST1	POST2	0.36 (0.33)	0.33 (0.38)			
				POST1	POST3	0.36 (0.33)	0.4 (0.34)			
				POST2	POST3	0.33 (0.38)	0.4 (0.34)			

SUPPLEMENTAL FILE 1. Continue

Variables	Field position	Friedman Statistic	p	Season 1	Season 2	Median1 (IQR 1)	Median 2 (IQR 2)	Wilcoxon Statistic	p adjusted	95%CI
Maximum speed (km/h)	OVERALL	9.8	**0.044**	PRE	INJ	33.3 (1.91)	32.96 (2.42)	155	0.249	0.01; 1.16
				PRE	POST1	33.3 (1.91)	32.67 (1.93)	119	**0.033**	0.10; 1.16
				PRE	POST2	33.3 (1.91)	33 (1.73)	117	**0.020**	0.16; 1.07
				PRE	POST3	33.3 (1.91)	32.78 (2.05)	181	0.464	0.08; 0.83
				INJ	POST1	32.96 (2.42)	32.67 (1.93)	274	1.000	-0.36; 1.29
				INJ	POST2	32.96 (2.42)	33 (1.73)	259.5	1.000	-0.51; 0.57
				INJ	POST3	32.96 (2.42)	32.78 (2.05)	279.5	1.000	-0.78; 0.62
				POST1	POST2	32.67 (1.93)	33 (1.73)	257.5	1.000	-0.58; 0.33
				POST1	POST3	32.67 (1.93)	32.78 (2.05)	183	0.503	-0.85; -0.1
				POST2	POST3	33 (1.73)	32.78 (2.05)	222	1.000	-0.43; 0.26
	≤ 25 YEARS	3.33	0.505	PRE	INJ	33.34 (2)	32.56 (2.61)			
				PRE	POST1	33.34 (2)	33.01 (2.08)			
				PRE	POST2	33.34 (2)	33.12 (1.7)			
				PRE	POST3	33.34 (2)	33.01 (1.77)			
				INJ	POST1	32.56 (2.61)	33.01 (2.08)			
				INJ	POST2	32.56 (2.61)	33.12 (1.7)			
				INJ	POST3	32.56 (2.61)	33.01 (1.77)			
				POST1	POST2	33.01 (2.08)	33.12 (1.7)			
				POST1	POST3	33.01 (2.08)	33.01 (1.77)			
				POST2	POST3	33.12 (1.7)	33.01 (1.77)			
	> 25 YEARS	13.11	**0.011**	PRE	INJ	33 (1.83)	33.33 (1.62)	23	1.000	-0.3; 1.23
				PRE	POST1	33 (1.83)	32.32 (1.67)	6	0.058	0.26; 2.51
				PRE	POST2	33 (1.83)	32.31 (1.7)	6	**0.017**	0.4; 1.85
				PRE	POST3	33 (1.83)	31.8 (1.54)	4	**0.009**	0.28; 1.7
				INJ	POST1	33.33 (1.62)	32.32 (1.67)	30	1.000	-0.3; 2.36
				INJ	POST2	33.33 (1.62)	32.31 (1.7)	32	1.000	-0.28; 1.64
				INJ	POST3	33.33 (1.62)	31.8 (1.54)	35	1.000	-0.63; 1.93
				POST1	POST2	32.32 (1.67)	32.31 (1.7)	44	1.000	-0.75; 0.82
				POST1	POST3	32.32 (1.67)	31.8 (1.54)	39	1.000	-0.83; 0.54
				POST2	POST3	32.31 (1.7)	31.8 (1.54)	43	1.000	-0.88; 0.72

SUPPLEMENTAL FILE 2. Match data before (PRE), the season of the injury (INJ) and up to three seasons (POST1, POST2, POST3, respectively) after the RTP in professional football players with primary anterior cruciate ligament injury depending on their field position (n=34).

Variables	Field position	Friedman Statistic	p	Season 1	Season 2	Median1 (IQR 1)	Median 2 (IQR 2)	Wilcoxon Statistic	p	p adjusted	95%CI
Minutes of match play (min)	DEF	17.54	**0.002**	PRE	INJ	2633.98 (1061.87)	870.54 (1332.2)	6	**0.002**	**0.017**	477.63; 2191.13
				PRE	POST1	2633.98 (1061.87)	1352.13 (1011.3)	8	**0.003**	**0.031**	329.54; 1653.13
				PRE	POST2	2633.98 (1061.87)	2189.32 (995.79)	17	**0.025**	0.245	176.24; 971.45
				PRE	POST3	2633.98 (1061.87)	1758.08 (1484.44)	23	0.068	0.676	-351.96; 1608.78
				INJ	POST1	870.54 (1332.2)	1352.13 (1011.3)	35	0.296	1.000	-1284.51; 184.75
				INJ	POST2	870.54 (1332.2)	2189.32 (995.79)	15	**0.017**	0.166	-1291.47; -91.76
				INJ	POST3	870.54 (1332.2)	1758.08 (1484.44)	32	0.217	1.000	-1658.85; 340.77
				POST1	POST2	1352.13 (1011.3)	2189.32 (995.79)	31	0.194	1.000	-1199.82; 68.1
				POST1	POST3	1352.13 (1011.3)	1758.08 (1484.44)	36	0.326	1.000	-980.49; 238.21
				POST2	POST3	2189.32 (995.79)	1758.08 (1484.44)	46	0.715	1.000	-544.5; 538.37
	MID	6.16	0.187	PRE	INJ	499.16 (1280.53)	325.6 (461.65)				
				PRE	POST1	499.16 (1280.53)	1122.36 (1087.86)				
				PRE	POST2	499.16 (1280.53)	841.09 (1038.84)				
				PRE	POST3	499.16 (1280.53)	1912.79 (1758.56)				
				INJ	POST1	325.6 (461.65)	1122.36 (1087.86)				
				INJ	POST2	325.6 (461.65)	841.09 (1038.84)				
				INJ	POST3	325.6 (461.65)	1912.79 (1758.56)				
				POST1	POST2	1122.36 (1087.86)	841.09 (1038.84)				
				POST1	POST3	1122.36 (1087.86)	1912.79 (1758.56)				
				POST2	POST3	841.09 (1038.84)	1912.79 (1758.56)				
	FOR	16.33	**0.003**	PRE	INJ	1982.54 (1107.05)	763.55 (835.88)	0	**< 0.001**	**0.005**	431.57; 1546.38
				PRE	POST1	1982.54 (1107.05)	1007.42 (1445.09)	16	0.077	0.771	-157.97; 1675.8
				PRE	POST2	1982.54 (1107.05)	1960.55 (1332.96)	33	0.677	1.000	-409.41; 378.36
				PRE	POST3	1982.54 (1107.05)	1007.3 (910.35)	11	0.027	0.269	76.59; 1486.14
				INJ	POST1	763.55 (835.88)	1007.42 (1445.09)	32	0.622	1.000	-1238.43; 980.56
				INJ	POST2	763.55 (835.88)	1960.55 (1332.96)	5	**0.005**	**0.049**	-1568.52; -314.7
				INJ	POST3	763.55 (835.88)	1007.3 (910.35)	17	0.092	0.923	-940.66; 29.39
				POST1	POST2	1007.42 (1445.09)	1960.55 (1332.96)	16	0.077	0.771	-1527.75; 65.08
				POST1	POST3	1007.42 (1445.09)	1007.3 (910.35)	39	1.000	1.000	-637.7; 513.99
				POST2	POST3	1960.55 (1332.96)	1007.3 (910.35)	12	**0.034**	0.342	-0.11; 1261.69

SUPPLEMENTAL FILE 2. Continue

Variables	Field position	Friedman Statistic	p	Season 1	Season 2	Median1 (IQR 1)	Median 2 (IQR 2)	Wilcoxon Statistic	p	p adjusted	95%CI
Total distance/min (m/min)	DEF	1.54	0.819	PRE	INJ	104.57 (100.55)	106.97 (98.33)				
				PRE	POST1	104.57 (100.55)	104.38 (108.52)				
				PRE	POST2	104.57 (100.55)	148.16 (104.21)				
				PRE	POST3	104.57 (100.55)	103.4 (107.06)				
				INJ	POST1	106.97 (98.33)	104.38 (108.52)				
				INJ	POST2	106.97 (98.33)	148.16 (104.21)				
				INJ	POST3	106.97 (98.33)	103.4 (107.06)				
				POST1	POST2	104.38 (108.52)	148.16 (104.21)				
				POST1	POST3	104.38 (108.52)	103.4 (107.06)				
				POST2	POST3	148.16 (104.21)	103.4 (107.06)				
	MID	7.12	0.13	PRE	INJ	127.97 (38.48)	128.31 (38.89)				
				PRE	POST1	127.97 (38.48)	131.63 (114.55)				
				PRE	POST2	127.97 (38.48)	128.69 (44.07)				
				PRE	POST3	127.97 (38.48)	121.97 (33.52)				
				INJ	POST1	128.31 (38.89)	131.63 (114.55)				
				INJ	POST2	128.31 (38.89)	128.69 (44.07)				
				INJ	POST3	128.31 (38.89)	121.97 (33.52)				
				POST1	POST2	131.63 (114.55)	128.69 (44.07)				
				POST1	POST3	131.63 (114.55)	121.97 (33.52)				
				POST2	POST3	128.69 (44.07)	121.97 (33.52)				
	FOR	4.87	0.301	PRE	INJ	109.58 (8.82)	112.19 (32.51)				
				PRE	POST1	109.58 (8.82)	118.04 (32.59)				
				PRE	POST2	109.58 (8.82)	112.19 (15.69)				
				PRE	POST3	109.58 (8.82)	112.3 (18.75)				
				INJ	POST1	112.19 (32.51)	118.04 (32.59)				
				INJ	POST2	112.19 (32.51)	112.19 (15.69)				
				INJ	POST3	112.19 (32.51)	112.3 (18.75)				
				POST1	POST2	118.04 (32.59)	112.19 (15.69)				
				POST1	POST3	118.04 (32.59)	112.3 (18.75)				
				POST2	POST3	112.19 (15.69)	112.3 (18.75)				

SUPPLEMENTAL FILE 2. Continue

Variables	Field position	Friedman Statistic	p	Season 1	Season 2	Median1 (IQR 1)	Median 2 (IQR 2)	Wilcoxon Statistic	p	p adjusted	95%CI
Sprint distance/min (m/min)	DEF	2.69	0.612	PRE	INJ	6.73 (6.32)	6.84 (4.64)				
				PRE	POST1	6.73 (6.32)	6.39 (3.52)				
				PRE	POST2	6.73 (6.32)	8.48 (6.89)				
				PRE	POST3	6.73 (6.32)	4.18 (7.06)				
				INJ	POST1	6.84 (4.64)	6.39 (3.52)				
				INJ	POST2	6.84 (4.64)	8.48 (6.89)				
				INJ	POST3	6.84 (4.64)	4.18 (7.06)				
				POST1	POST2	6.39 (3.52)	8.48 (6.89)				
				POST1	POST3	6.39 (3.52)	4.18 (7.06)				
				POST2	POST3	8.48 (6.89)	4.18 (7.06)				
	MID	4.90	0.298	PRE	INJ	6.52 (2.87)	6.06 (2.72)				
				PRE	POST1	6.52 (2.87)	7.52 (5.76)				
				PRE	POST2	6.52 (2.87)	7.36 (4.34)				
				PRE	POST3	6.52 (2.87)	5.16 (5.21)				
				INJ	POST1	6.06 (2.72)	7.52 (5.76)				
				INJ	POST2	6.06 (2.72)	7.36 (4.34)				
				INJ	POST3	6.06 (2.72)	5.16 (5.21)				
				POST1	POST2	7.52 (5.76)	7.36 (4.34)				
				POST1	POST3	7.52 (5.76)	5.16 (5.21)				
				POST2	POST3	7.36 (4.34)	5.16 (5.21)				
	FOR	12.47	**0.014**	PRE	INJ	8.49 (1.81)	9.36 (4.73)	21	0.176	1.000	-1.78; 0.28
				PRE	POST1	8.49 (1.81)	9.3 (5.22)	25	0.301	1.000	-3.42; 0.52
				PRE	POST2	8.49 (1.81)	9.08 (3.26)	37	0.91	1.000	-1.53; 1.6
				PRE	POST3	8.49 (1.81)	4.84 (4.51)	24	0.266	1.000	-0.62; 4.97
				INJ	POST1	9.36 (4.73)	9.3 (5.22)	28	0.424	1.000	-1.17; 1.8
				INJ	POST2	9.36 (4.73)	9.08 (3.26)	22	0.204	1.000	-0.61; 2.56
				INJ	POST3	9.36 (4.73)	4.84 (4.51)	12	**0.034**	0.342	1.13; 5.43
				POST1	POST2	9.3 (5.22)	9.08 (3.26)	30	0.519	1.000	-1.18; 1.74
				POST1	POST3	9.3 (5.22)	4.84 (4.51)	10	**0.021**	0.210	1.62; 4.37
				POST2	POST3	9.08 (3.26)	4.84 (4.51)	8	**0.012**	0.122	0.07; 3.67

SUPPLEMENTAL FILE 2. Continue

Variables	Field position	Friedman Statistic	p	Season 1	Season 2	Median1 (IQR 1)	Median 2 (IQR 2)	Wilcoxon Statistic	p	p adjusted	95%CI
Sprints/min (n/min)	DEF	2.571	0.632	PRE	INJ	0.41 (0.35)	0.39 (0.34)				
				PRE	POST1	0.41 (0.35)	0.37 (0.26)				
				PRE	POST2	0.41 (0.35)	0.49 (0.47)				
				PRE	POST3	0.41 (0.35)	0.41 (0.38)				
				INJ	POST1	0.39 (0.34)	0.37 (0.26)				
				INJ	POST2	0.39 (0.34)	0.49 (0.47)				
				INJ	POST3	0.39 (0.34)	0.41 (0.38)				
				POST1	POST2	0.37 (0.26)	0.49 (0.47)				
				POST1	POST3	0.37 (0.26)	0.41 (0.38)				
				POST2	POST3	0.49 (0.47)	0.41 (0.38)				
	MID	2.2	0.699	PRE	INJ	0.42 (0.32)	0.38 (0.17)				
				PRE	POST1	0.42 (0.32)	0.43 (0.37)				
				PRE	POST2	0.42 (0.32)	0.46 (0.31)				
				PRE	POST3	0.42 (0.32)	0.38 (0.23)				
				INJ	POST1	0.38 (0.17)	0.43 (0.37)				
				INJ	POST2	0.38 (0.17)	0.46 (0.31)				
				INJ	POST3	0.38 (0.17)	0.38 (0.23)				
				POST1	POST2	0.43 (0.37)	0.46 (0.31)				
				POST1	POST3	0.43 (0.37)	0.38 (0.23)				
				POST2	POST3	0.46 (0.31)	0.38 (0.23)				
	FOR	6.133	0.189	PRE	INJ	0.47 (0.07)	0.52 (0.29)				
				PRE	POST1	0.47 (0.07)	0.54 (0.21)				
				PRE	POST2	0.47 (0.07)	0.51 (0.17)				
				PRE	POST3	0.47 (0.07)	0.52 (0.24)				
				INJ	POST1	0.52 (0.29)	0.54 (0.21)				
				INJ	POST2	0.52 (0.29)	0.51 (0.17)				
				INJ	POST3	0.52 (0.29)	0.52 (0.24)				
				POST1	POST2	0.54 (0.21)	0.51 (0.17)				
				POST1	POST3	0.54 (0.21)	0.52 (0.24)				
				POST2	POST3	0.51 (0.17)	0.52 (0.24)				

SUPPLEMENTAL FILE 2. Continue

Variables	Field position	Friedman Statistic	p	Season 1	Season 2	Median1 (IQR 1)	Median 2 (IQR 2)	Wilcoxon Statistic	p	p adjusted	95%CI
Maximum speed (km/h)	DEF	5.022	0.285	PRE	INJ	32.99 (1.57)	33.06 (2.23)				
				PRE	POST1	32.99 (1.57)	32.15 (1.25)				
				PRE	POST2	32.99 (1.57)	32.24 (1.58)				
				PRE	POST3	32.99 (1.57)	32.09 (1.4)				
				INJ	POST1	33.06 (2.23)	32.15 (1.25)				
				INJ	POST2	33.06 (2.23)	32.24 (1.58)				
				INJ	POST3	33.06 (2.23)	32.09 (1.4)				
				POST1	POST2	32.15 (1.25)	32.24 (1.58)				
				POST1	POST3	32.15 (1.25)	32.09 (1.4)				
				POST2	POST3	32.24 (1.58)	32.09 (1.4)				
	MID	0.679	0.954	PRE	INJ	32.43 (1.53)	30.97 (2.86)				
				PRE	POST1	32.43 (1.53)	32.35 (2.11)				
				PRE	POST2	32.43 (1.53)	31.99 (2.66)				
				PRE	POST3	32.43 (1.53)	31.93 (1.23)				
				INJ	POST1	30.97 (2.86)	32.35 (2.11)				
				INJ	POST2	30.97 (2.86)	31.99 (2.66)				
				INJ	POST3	30.97 (2.86)	31.93 (1.23)				
				POST1	POST2	32.35 (2.11)	31.99 (2.66)				
				POST1	POST3	32.35 (2.11)	31.93 (1.23)				
				POST2	POST3	31.99 (2.66)	31.93 (1.23)				
	FOR	9.506	0.052	PRE	INJ	34.39 (1.44)	33.88 (1.25)				
				PRE	POST1	34.39 (1.44)	33.32 (1.68)				
				PRE	POST2	34.39 (1.44)	33.46 (1.11)				
				PRE	POST3	34.39 (1.44)	33.5 (1.12)				
				INJ	POST1	33.88 (1.25)	33.32 (1.68)				
				INJ	POST2	33.88 (1.25)	33.46 (1.11)				
				INJ	POST3	33.88 (1.25)	33.5 (1.12)				
				POST1	POST2	33.32 (1.68)	33.46 (1.11)				
				POST1	POST3	33.32 (1.68)	33.5 (1.12)				
				POST2	POST3	33.46 (1.11)	33.5 (1.12)				

DEF: defender; MID: midfielder; FOR: forward; IQR: interquartile range; CI: confidence interval. Significant statistical differences are indicated in bold.
